# Role of astrocytes in the pathogenesis of perinatal brain injury

**DOI:** 10.1186/s10020-025-01328-w

**Published:** 2025-08-13

**Authors:** Ling Li, Xuewei Cui, Baoli Zhu, Lele Zhou, Yaya Guo, Tianjing Liu, Yongyan Shi

**Affiliations:** 1 Department of Pediatrics, Shengjing Hospital of China Medical University, Shenyang, 110004 China; 2 Department of Neonatology, Tacheng Hospital of China Medical University, Tacheng, 834300 China

**Keywords:** Astrocytes, Perinatal brain injury, JAK/STAT, Notch, NF-κB, Glutamate transporter

## Abstract

Astrocytes, the most abundant glial cells in the central nervous system (CNS), play critical roles in blood–brain barrier (BBB) maintenance, synaptogenesis, neurotransmission, and metabolic regulation. In response to perinatal brain injury, astrocytes release inflammatory mediators that drive neuroinflammation, disrupting normal brain development. Key signaling pathways, including Janus kinase/signal transducers and activators of transcription (JAK/STAT), nuclear factor kappa B (NF-κB), Notch, and glutamate transporter signaling, are activated during this process, contributing to astrocyte dysfunction and neuronal damage. Astrocytes also engage in dynamic crosstalk with microglia, oligodendrocytes, and neurons, further influencing the injury response. Biomarkers such as glial fibrillary acidic protein (GFAP) and calcium-binding protein (S100β) highlight astrocyte activation and its role in pathology. By targeting these signaling pathways and glial interactions, novel therapeutic strategies can be developed to mitigate neurodevelopmental and perinatal brain injuries associated with astrocyte dysfunction.

**Graphic abstract:**

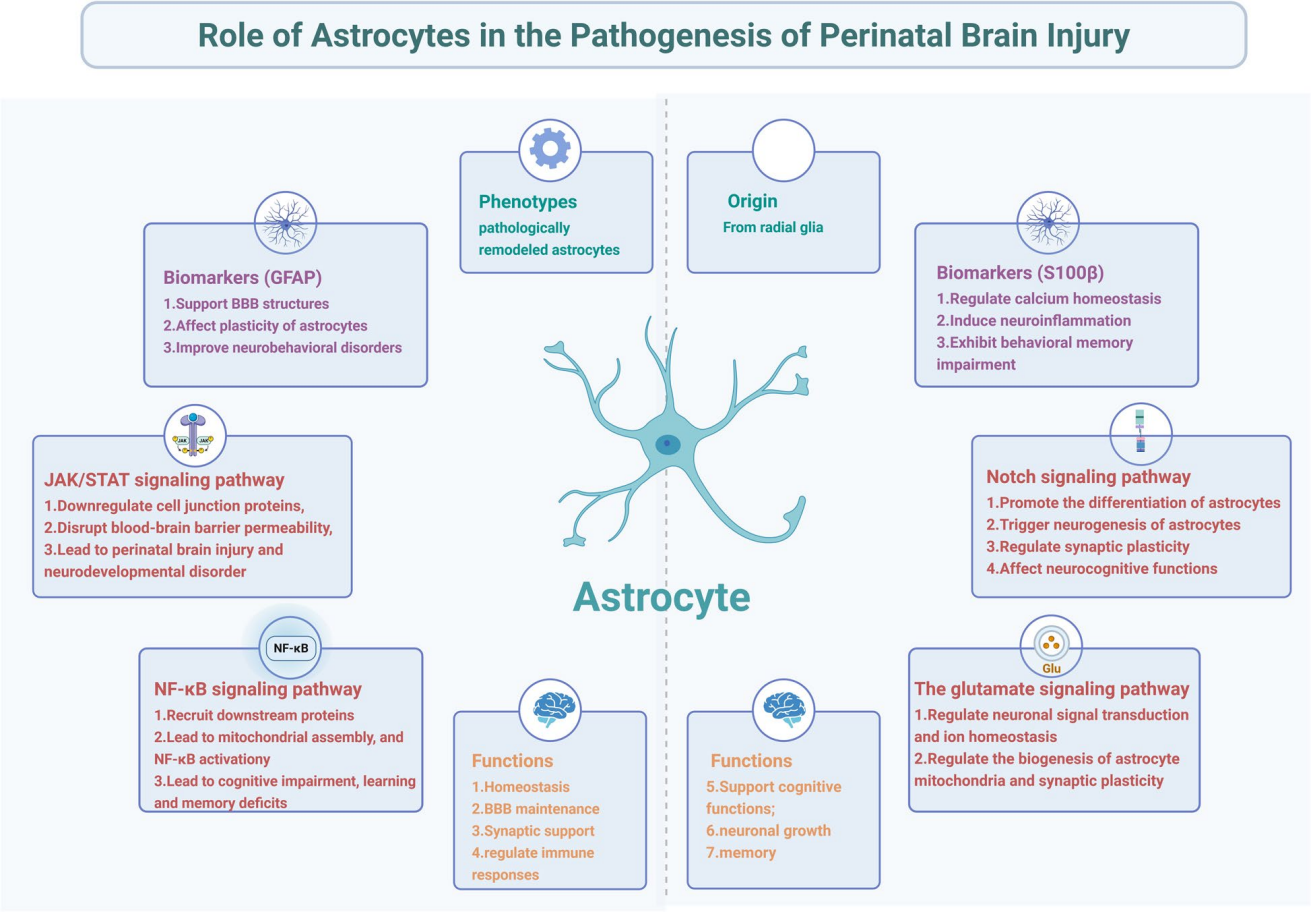

## Introduction

Preterm birth has a global population of approximately 15 million (Liu et al. 2016). It is associated with a high incidence and mortality of complications, such as hypoxic–ischemic encephalopathy (HIE), intraventricular hemorrhage, periventricular leukomalacia, perinatal stroke, cerebral palsy (Beeraka et al. 2022; Purcell et al. 2023; Razak, Patel, Durrani and Pullattayil, 2023). Perinatal brain injury is associated with exposure to various perinatal inflammatory triggers, including prenatal infections (e.g., chorioamnionitis), hypoxia–ischemia (HI), and postnatal injury triggers (e.g., oxidative stress and sepsis) (Herz et al. 2022; Reiss et al. 2022). The pathogenesis is complex, involving fetal inflammation, neonatal hypoxia/ischemia, ischemia–reperfusion, brain immune cell activation, excitotoxicity, and free radical production (Ophelders et al., 2020), leading to severe long-term neurodevelopmental deficits (Calado et al. 2023; Leavy & Jimenez Mateos, 2020).

Astrocytes make the largest class of neuroglial cells that are involved in neurogenesis, synaptogenesis, and neuronal functions during the perinatal period (Diaz-Castro, Bernstein, Coppola, Sofroniew and Khakh, 2021; Felix et al. 2021). They can also influence the neuronal microenvironment by secreting or removing factors such as vascular endothelial growth factor (VEGF), transforming growth factor-β (TGF-β), glial cell-derived neurotrophic factor (GDNF), and brain-derived neurotrophic factor (BDNF), which act on receptors on neurons, glia, and blood vessels (Guo and Bi 2024). Additionally, astrocytes interact with the endothelium to regulate the development and function of the BBB (Purnell et al. 2023). Furthermore, astrocytes produce antioxidant and anti-inflammatory proteins, which help neurons communicate with one another correctly, and support neuronal growth and advanced cognitive functions such as repair and memory (Li et al. 2018; Talifu et al. 2023; Valles et al., 2023). Moreover, astrocytes maintain glutamate homeostasis primarily through high-affinity glutamate transporters (GLT-1/EAAT2 and GLAST/EAAT1), which rapidly clear synaptic glutamate to prevent excitotoxicity, while also supporting mitochondrial function and redox balance to protect against neuronal injury (Chandrasekaran et al. 2021; Dabrowska et al. 2018; Jimenez-Blasco et al. 2023).

This article reviews the origin, differentiation, phenotypes, and biomarkers of astrocytes and their role in perinatal brain injuries, with emphasis on the signaling pathways activated by astrocytes in perinatal brain injuries. Then, we outline the crosstalk between astrocytes and microglia, oligodendrocytes, and neurons in perinatal brain injury. This review aims to provide a profound insight into the underlying mechanisms of astrocyte-induced perinatal brain injuries.

## Overview of astrocytes

### Origin and differentiation of astrocytes

Almost all neurons and most glial cells in the developing brain originate from neuroepithelial progenitor cells in the subventricular zone (SVZ) of the embryonic nervous system, particularly those in the extended external SVZ. Progenitor cells can migrate radially from the SVZ, producing clusters of astrocytes in the same cortical column (Torres-Ceja and Olsen 2022). During mid-gestation, SVZ progenitors generate white matter astrocytes that exhibit robust proliferative responses to growth factors and enhanced oxidative stress resistance, while VZ progenitors produce gray matter astrocytes with superior calcium signaling and synaptic modulation capacities—functional differences attributed to their distinct transcriptional programs and niche-sptecific extracellular cues (Allen et al. 2022).

Astrocyte development begins during mid-gestation and continues into the early postnatal period, with critical phases of proliferation, migration, and differentiation occurring during the third trimester of pregnancy. This timeline is particularly relevant to perinatal brain injury, as preterm birth often disrupts these developmental processes. For instance, preterm infants born before the completion of astrocyte maturation (typically around 24–32 weeks of gestation) may exhibit impaired astrocyte migration and differentiation, leading to abnormal cortical organization and disrupted BBB formation (Díaz-Castro et al. 2023). Preterm birth can also expose the developing brain to inflammatory and HI insults, which further impair astrocyte function and exacerbate perinatal brain injury (Van Steenwinckel et al. 2024).

Cell differentiation generally involves the asymmetric division of stem cells to produce stem cells and progenitors, as well as the symmetric division of neural progenitor cells into a progenitor pool, which then transform into radial neuroglia cells (Obernier and Alvarez-Buylla, 2019). Radial glial cells give rise to precursor astrocytes by asymmetric division and can also directly transform into astrocytes (Chen, Zheng, Huang, Ma, and Li, 2022; Yeh, Wu, Huang, and Verkhratsky, 2023). These astrocytes migrate toward the cortex, with astrocyte endothelin-1 overexpression promoting both progenitor cell proliferation and astrocyte differentiation via the Jak2/Stat3 pathway (Cheng et al. 2019). However, in the context of preterm birth, this migration can be disrupted by inflammation or hypoxia, leading to mislocalized astrocytes and impaired cortical development.

Throughout the CNS, differentiated astrocyte populations align into morphological domains, inducing the diversity of astrocytes (Villarreal and Vogel, 2021). This diversity is essential for normal brain function, but preterm birth can alter astrocyte heterogeneity, particularly in vulnerable regions like the periventricular white matter. In preterm infants, HI injury can lead to reactive astrogliosis, characterized by hypertrophic astrocytes that contribute to neuroinflammation and further neuronal damage (Fang et al. 2024).

In summary, the timeline of astrocyte development is closely linked to the risk of perinatal brain injury in preterm infants. Disruptions in astrocyte proliferation, migration, and differentiation due to preterm birth or associated insults can have long-lasting consequences for brain development and function. Understanding these processes is critical for developing targeted interventions to mitigate perinatal brain injury.

### Astrocyte phenotypes

Astrocytes are distributed in all nervous system regions and categorized into multiple phenotypes based on their location and function. They are classified into subtypes including (but not limited to) protoplasmic astrocytes and fibrillar astrocytes found in gray and white matter (Baldwin 2022; Yoon, Walters, Paulsen, and Scarisbrick, 2017). Protoplasmic astrocytes exhibit a complex morphology with extensive branches within their structural domains, which may distribute in a globular pattern, engage in many synapses, and wrap blood vessels with their ends. On the contrary, fibrillar astrocytes are organized along white matter bundles, characterized by straight and long protrusions with few branching points. These protrusions are aligned with myelinated axons and contribute to maintaining homeostasis (Köhler et al. 2021).

In addition, astrocytes can undergo significant changes in response to injury, transitioning into reactive astrocytes. In response to injury or disease, astrocytes undergo dynamic remodeling, traditionally classified into “A1” (neurotoxic) and “A2” (neuroprotective) phenotypes. However, recent consensus guidelines (Escartin et al. 2021) emphasize that this binary framework oversimplifies astrocyte reactivity, which is instead context-dependent and shaped by diverse molecular and epigenetic reprogramming events. Instead, reactive astrocytes are now recognized as undergoing complex pathological remodeling, influenced by microenvironmental cues such as microglial activation, metabolic stress, and epigenetic reprogramming (Pavlou, Grandbarbe, Buckley, Niclou, and Michelucci, 2019). In fact, it is better to consider that neurotoxic astrocytes are pathologically remodeled astrocytes. For instance, in lipopolysaccharide (LPS)-induced neuroinflammation in neonatal models, astrocytes exhibit a pathogenic phenotype characterized by sustained pro-inflammatory gene expression and epigenetic modifications (e.g., altered DNA methylation and histone acetylation) that drive persistent neurotoxicity (Cuautle, Donna, Cieri, Villarreal, & Ramos, 2024). This reprogramming can impair synaptic support and exacerbate neuronal damage, rather than fitting into rigid A1/A2 categories. Thus, future research should focus on defining astrocyte reactivity based on functional states rather than dichotomous classifications, while also exploring epigenetic and metabolic drivers of pathogenic remodeling for targeted therapeutic interventions.

Beyond the reactive subtypes, several specialized astrocyte populations exist in human, including villous astrocytes and radial astrocytes in the cerebellum, pituitary cells in the pituitary gland, perivascular and limbic astrocytes, Gomori-positive astrocytes, and surface-associated astrocytes (Cohen and Torres 2019; Westergard and Rothstein 2020). These astrocytes exhibit unique properties and perform distinct functions that contribute to the overall maintenance and defense of the CNS. Perivascular astrocytes are integral to the regulation of the BBB, where they interact with endothelial cells to modulate vascular tone and nutrient transport (Pfau et al. 2024). Radial astrocytes in the cerebellum play a crucial role in guiding neuronal migration during development, while pituitary cells in the pituitary gland are involved in neuroendocrine signaling and hormone regulation (de Reus et al. 2024). Limbic astrocytes are important in modulating synaptic plasticity and emotional behavior, highlighting their role in higher-order brain functions (Martínez-Gallego, Coatl-Cuaya, and Rodriguez-Moreno, 2024). Gomori-positive astrocytes and surface-associated astrocytes contribute to structural support and the regulation of extracellular ion and neurotransmitter balance.

These specialized astrocytes, along with their classic and reactive counterparts, maintain CNS homeostasis by providing metabolic support to neurons, regulating synaptic activity, and defending against pathological insults. For instance, they participate in the uptake and recycling of neurotransmitters such as glutamate, preventing excitotoxicity, and they release neurotrophic factors that promote neuronal survival and repair (Lee et al. 2022). Furthermore, astrocytes play a critical role in responding to injury or disease by modulating inflammation, scar formation, and tissue remodeling. Their remarkable heterogeneity and functional adaptability enable them to play diverse roles across different brain regions and under various physiological and pathological conditions (Giovannoni and Quintana 2020).

### The role of astrocytes in perinatal brain injuries

Astrocytes, as structural components of the neurovascular unit, play a critical role in maintaining brain homeostasis and neuronal function. They extend peripheral projections that not only support and segregate neuronal cells but also release pro- or anti-angiogenic factors, regulating angiogenesis and contributing to the structure and function of BBB (Puebla, Tapia, and Espinoza, 2022). Additionally, astrocytes are essential for synapse formation and maintenance, synaptic plasticity, and the uptake and recycling of glutamate, which is crucial for preventing excitotoxicity (Endo et al., 2022; Qian, Qin, Lai, Zhang, and Zhang, 2023).

#### Astrocyte dysfunction in perinatal brain injury

Astrocyte dysfunction is a hallmark of perinatal brain injury, contributing to both acute and long-term neurological deficits. Examples of astrocyte dysfunction in perinatal brain injury are listed in Table 1.

**Table 1:** Astrocyte dysfunctions in perinatal brain injury

Mechanism	Pathological effect	Example study/model	References
Glutamate transporter deficiency	Neuronal excitotoxicity and cell death	Hypoxia–ischemia model in neonatal rats	(Goodrich et al. 2013)
Reactive astrogliosis	Neuroinflammation and BBB disruption	Intrauterine growth retardation model in piglets	(Wixey et al. 2019)
Mitochondrial dysfunction	Impaired energy production and oxidative stress	Hypoxia–ischemia model in neonatal rats	(Berger et al. 2016)
Inflammatory signaling	Microglial activation and white matter injury	Cerebral Palsy model of premature Infants in rats	(Liu, Shen, Plane and Deng, 2013)
Synaptic support disruption	Cognitive and motor deficits	Hypoxia–ischemia model in neonatal rats	(Lai et al. 2011)

*BBB* blood–brain barrier

##### Glutamate excitotoxicity

In perinatal HIE, immature astrocytes fail to express adequate glutamate transporters such as GLT-1 (EAAT2), leading to excessive extracellular glutamate accumulation (Razak and Hussain 2019). This results in neuronal excitotoxicity and cell death. It has been shown that enhancing glutamate transporter protein 1 (GLT-1) expression, for example through ceftriaxone treatment, can mitigate neuronal damage in neonatal rat models of HIE (Goodrich et al. 2013).

##### Reactive astrogliosis

Following perinatal brain injury, astrocytes undergo reactive astrogliosis, adopting either neurotoxic or neuroprotective phenotypes. The inflammatory environment of preterm injury strongly skews the balance toward the pathologically remodeled astrocytes (Jung and Ryu 2023), marked by upregulation of pro-inflammatory cytokines (TNF-α, IL-1β, IL-6) and complement genes, which exacerbate BBB disruption and neuronal damage (Wixey et al. 2019). This predominance of pathologically remodeled astrocytes contributes to white matter injury and long-term neurological impairments in preterm infants (Mallard et al. 2014; Roessmann & Gambetti 1986).

##### Mitochondrial dysfunction

Astrocytes in the immature brain are particularly vulnerable to ischemic injury due to their reliance on mitochondrial metabolism. In neonatal HI models, astrocyte mitochondrial dysfunction leads to impaired energy production and increased oxidative stress, further exacerbating neuronal injury (Berger et al. 2016).

##### Inflammation and microglial activation

Astrocytes interact closely with microglia in response to perinatal brain injury. Astrocyte-derived ATP release through connexin hemichannels triggers microglial activation, propagating inflammation and white matter injury (Dufour et al. 2024; Köhler et al. 2023; Liu et al. 2013). This inflammatory cascade is a key contributor to the pathogenesis of cerebral palsy in preterm infants.

##### Synaptic dysfunction

Astrocyte dysfunction disrupts synaptic maintenance and plasticity, leading to cognitive and motor deficits. In neonatal rat models of HI, astrocyte-mediated synaptic support is compromised, resulting in long-term neurological impairments (Lai et al. 2011).

### Astrocyte-mediated biomarkers during perinatal brain injuries

Intermediate filament GFAP and calcium-binding protein (S100β), expressed by two major genes controlling astrocyte differentiation, can be used as biomarkers to predict and diagnose perinatal brain injury (Menéndez-Valladares et al. 2020) (Table 2).

**Table 2:** Functions of biomarkers GFAP and S100β for astrocytes in perinatal brain injury

Functions	References
*GFAP*	
Support blood–brain barrier structures	( Jurga, Paleczna, Kadluczka, and Kuter, 2021 )
Affect the morphological and functional plasticity of astrocytes	(Lin, Yang, Chang, and Perng, 2021 )
Improve neonatal HIE-induced neurobehavioral disorders	( Yang et al. 2019 )
*S100β*	
Regulate intracellular calcium homeostasis and participate in adjacent astrocyte gap junction channels	( Juneja et al. 2019; Ryczko et al. 2021 )
Induce overexpression of nitric oxide synthase (iNOS) and neuroinflammation	( Langeh and Singh 2021; Zanoletti et al. 2023 )
Exhibit behavioral memory impairment	( Mader et al., 2022; Mari et al. 2019 )

*GFAP* glial fibrillary acidic protein; *HIE* hypoxic-ischemic encephalopathy

#### Glial filament glial fibrillary acid protein

GFAP refers to intermediate filament type III proteins that is expressed in the astrocyte cytoskeleton. It is expressed differently in cells from different brain regions and different types of neuronal stem cells (Verde et al. 2023). The general function of GFAP is to support astrocytes and the structure of the BBB. It labels the extensive branches of white matter astrocytes and their cell bodies, making it an ideal marker for investigating the morphology of astrocytes (Jurga et al., 2021). Additionally, GFAP is included in the highly dynamic structures of cell migration, thus modulating the morphological and functional plasticity of astrocytes (Lin et al. 2021).

Upon perinatal brain injury, astrocytes are activated into reactive astrocytes, which exhibit characteristic upregulation of intermediate filament protein (nanofilament) GFAP or nestin, increased immune reactivity, and the formation of neuroglial scars (Liu, Liu, Bao, Bai, and Wang, 2020). In a study using a neonatal HIE model, astrocyte activation could be regulated by transient receptor potential vanilloid 1 and its deficiency reduced the expression of GFAP and IL-1β by decreasing the phosphorylation of JAK2 and STAT3, which significantly reduced the neurobehavioral deficits in neonates (Yang et al. 2019). This implies that interventions targeting GFAP might alleviate the neuroinflammatory and astrocyte-mediated responses triggered by HI, thereby improving neurobehavioral outcomes in neonatal HIE. Furthermore, it has been demonstrated that fetal growth restriction is associated with an increased incidence and mortality of perinatal brain injury, as well as significantly elevated levels of GFAP, and NF-κB expression (Yawno et al. 2019; Yue et al. 2021). Besides, the expression of GFAP gene can be regulated by the JAK2 signal transducer, the STAT3 cascade, and NF-κB pathway (Liu, et al. 2020).

#### Calcium-binding protein (S100β)

S100β protein is a calcium-binding protein that is predominantly distributed in neuronal cells, including glial cells and Schwann cells. It is involved in intracellular calcium homeostasis, protein phosphorylation, enzyme activity, cell proliferation and differentiation, and cytoskeletal component dynamics. It also participates in gap junction channels between neighboring astrocytes, allowing intercellular communication in astrocyte networks (Ryczko et al. 2021). The calcium signaling can be transferred through gap junctions to form astrocyte syncytia, allowing communications over greater distances (Juneja et al. 2019). In addition, S100β leads to the induction of nitric oxide synthase overexpression. It is involved in neuroinflammatory processes that lead to cellular damage and apoptosis through activation of the mitogen-activated protein kinase pathway and disruption of the Wnt pathway and hyperphosphorylation of tau protein due to increased NF-κB expression (Langeh & Singh 2021; Zanoletti et al. 2023).

Fetuses and newborns are especially vulnerable to glial cell death caused by infections and oxidative stress due to their underdeveloped immune system. During the process of glial cell death or injury, the S100β protein released can cross the BBB and enter the brain, where it can be used as a marker for diagnosing brain damage, particularly for glial cell damage (Bersani et al. 2020; Perrone et al., 2023). According to previous research, adult male mice exposed to AQP4-IgG in utero had abnormal cortical vasculature, fewer dendritic spines in pyramidal and stellate neurons, more S100β + astrocytes in the inner olfactory cortex, as well as behavioral and memory deficits (Mader et al., 2022). In contrast, S100β inhibitor, by reducing S100β release, may protect against memory deficits and tissue damage, and promote astrocyte survival (Mari et al. 2019).

#### Clinical potential of GFAP and S100β as biomarkers in human perinatal brain injury

Growing clinical evidence supports the utility of GFAP and S100β as diagnostic and prognostic biomarkers in human neonates. Serum GFAP levels > 0.8 ng/mL within 6 h after birth show 82% sensitivity for predicting moderate-severe HIE, while cord blood S100β > 0.5 μg/L correlates with poor neurodevelopmental outcomes at 18–24 months (Caramelo et al. 2023; Zaigham et al. 2017). These biomarkers offer three key clinical advantages: early detection before symptom onset, quantitative monitoring of injury progression, and objective evaluation of therapeutic responses (Shi, Luo, Deol, & Tan, 2022; Zaigham, Lundberg, Hayes, Undén, and Olofsson, 2016). Current multicenter trials are validating standardized assays for their routine use in neonatal intensive care units, particularly for guiding therapeutic hypothermia decisions in HIE and predicting cerebral palsy risk in preterm infants (Chalak et al. 2014; Lv et al. 2015).

## Signaling pathways in astrocyte activation during perinatal brain injuries

Astrocytes are the most abundant type of glial cells in the CNS, mediating memory enhancement and actively participating in high-level brain functions. The expression of astrocyte receptors in vivo is regulated by neurochemical inputs, allowing astrocytes to sense specific signals in each area of the brain. During the embryonic period, astrocyte development is governed by multiple signaling pathways (including JAK-STAT, NF-κB, Notch, and high-affinity excitatory glutamate transporter pathways). Preclinical research has revealed the role of these pathways in the mechanisms underlying perinatal brain injuries.

### JAK/STAT signaling pathway

The JAK/STAT signaling pathway, a cytokine-stimulated pathway critical for CNS development, neuronal cell proliferation, survival, and differentiation, consists of three main components: tyrosine kinase-associated receptors, JAK kinases, and the transcription factor STAT (Brenner and Messing 2021). Tyrosine kinase-associated receptors bind to various cytokines and induce receptor dimerization. JAK kinases, which share extensive homology with tyrosine kinases, couple to and phosphorylate these receptors (Bharadwaj, Kasembeli, Robinson, and Tweardy, 2020). As an upstream kinase of the STAT pathway, JAK activation leads to the phosphorylation of STAT in the cytoplasm, recruiting STAT proteins with SH2 domains to the binding site (Rodriguez et al., 2023). Subsequently, STAT form homo- or heterodimers, translocate to the nucleus, and regulate gene transcription through specific DNA elements, completing cytokine receptor-mediated signaling (Ott, Faletti, Heeg, Andreani, & Grimbacher, 2023).

**Fig. 1:**
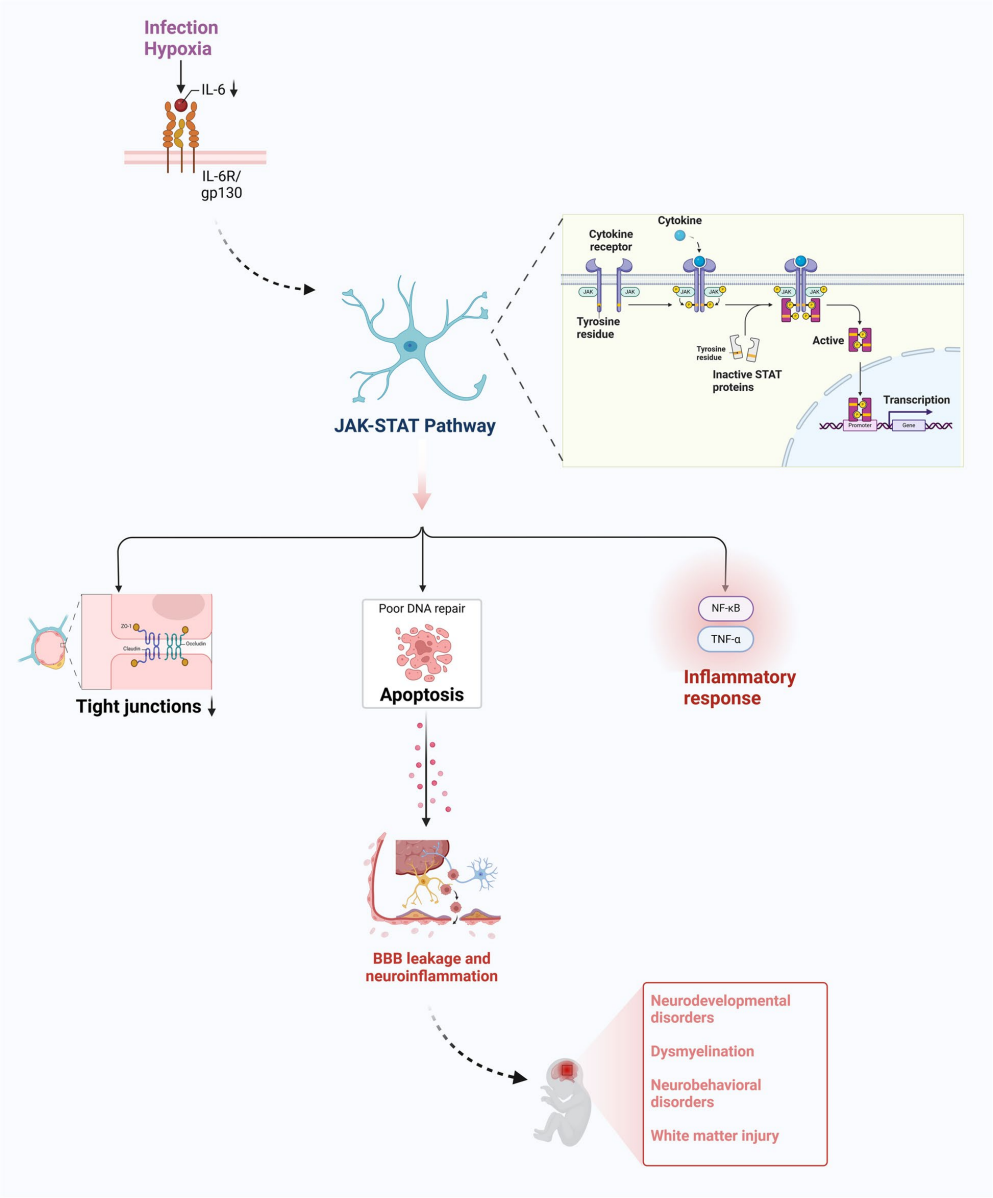
The JAK/STAT signaling pathway in astrocyte reactivity and the pathogenesis of perinatal brain injury. Infection and hypoxia can induce the production of interleukin-6, which in turn activates the JAK-STAT signaling pathway and susequently TNF-α signaling via NF-κB and cell apoptosis, participating in inflammatory response, downregulating cell junction proteins, disrupting blood–brain barrier permeability, leading to perinatal brain injury and neurodevelopmental disorders. *JAK/STAT* Janus kinase/signal transducers and activators of transcription; *TNF-α* tumour necrosis factor alpha; *NF-κB* nuclear factor-kappa B; *BBB* blood–brain barrier (Figure created with Biorender.com)

This pathway is extensively activated in the developing brain, with expression levels changing as the brain develops (Kumar et al. 2023) (Fig. 1). Matthew Raymond et al. (Raymond, Li, Mangin, Huntsman, and Gallo, 2011) demonstrates that chronic perinatal hypoxia reduces the function of the glutamate-aspartate transporter (GLAST) in astrocytes, which is essential for maintaining glutamate homeostasis and preventing excitotoxicity. Hypoxia-induced JAK/STAT pathway hyperactivation disrupts astrocyte function by: promoting excessive GFAP expression and reactive gliosis, impairing glutamate uptake through EAAT2 downregulation, and triggering pro-inflammatory cytokine release (IL-6, TNF-α) (M. Yang et al. 2022). These pathological changes directly contribute to white matter injury, as demonstrated in neonatal rodent models where JAK2/STAT3 inhibition preserved oligodendrocyte survival and myelination (X. M. Chen, Yu, Wang, Zhao, and Li, 2019). Additionally, JAK/STAT3 signaling plays a role in inflammatory responses through TNF-α signaling via NF-κB, regulates apoptosis (Wang et al. 2020; Weerasinghe-Mudiyanselage et al., 2022). These findings underscore the multifaceted role of the JAK/STAT pathway in CNS development, injury, and repair.

Several cytokines (IL-6, IL-10, and others) and growth factors (EGF, PDGF, CSF, EPO, and others) function by activating the JAK-STAT pathway and susequently induce astrocyte activation (Chen et al., 2020b; Martin et al. 2023). IL-6 binds to its receptor and forms a high-affinity complex with gp130 on the surface of target cells, which activates gp130 into a homodimer, and consequently activates the JAK/STAT pathway. IL-6 can also promote disruption of BBB permeability by activating the JAK/STAT pathway in endothelial cells and downregulating intercellular junction proteins (Aliyu et al. 2022; Couto et al. 2019; Rose-John, Jenkins, Garbers, Moll, & Scheller, 2023). This causes a large number of inflammatory factors to penetrate the BBB, resulting in perinatal brain damage and neurodevelopmental disorders. Therefore, inflammatory responses and perinatal-associated encephalopathy can be attenuated by blocking the cascade amplification response of inflammatory factors conducted by IL-6 in the future.

The JAK/STAT signaling pathway regulates numerous molecular switches and functional alterations in reactive astrocytes. In early life, decreased STAT3 activation adversely affects astrocyte production, impeding the development of BBB and thus interfering with neurotransmitter signaling in the developing brain (Supasai et al. 2021). Studies in mouse models have demonstrated that miR-181b, when upregulated by JAK2/STAT3 signaling, may directly target S1PR1, a key regulator of endothelial cell integrity and BBB stability. The downregulation of S1PR1 disrupts endothelial cell adhesion and tight junction formation, leading to increased BBB permeability. This mechanism contributes to the leakage of inflammatory mediators into the brain, exacerbating neuroinflammation and cognitive deficits (Chen et al. 2020a; Yao et al. 2022). By downregulating PKD1 and the JAK/STAT pathway, miR-31 attenuated the inflammatory response and oxidative stress-induced neuronal damage in mice with ischemic asphyxia (J. Li et al., 2020c). Furthermore, (Wu, Xue, Zhang, and Zhao, 2020) in a study conducted in white matter-injured neonatal mice, neuroprotective dexmedetomidine may alleviate neurobehavioral impairments and myelin formation deficits in early postnatal mice exposed to LPS by attenuating STAT3-mediated reactive astrocyte proliferation. Further investigation of the regulatory mechanisms of the JAK-STAT signaling pathway in perinatal brain injury and its interactions with other signaling pathways may provide valuable clues for preventing and treating perinatal brain injury (L. Wang et al., 2021b).

### NF-κB signaling pathway

**Fig. 2:**
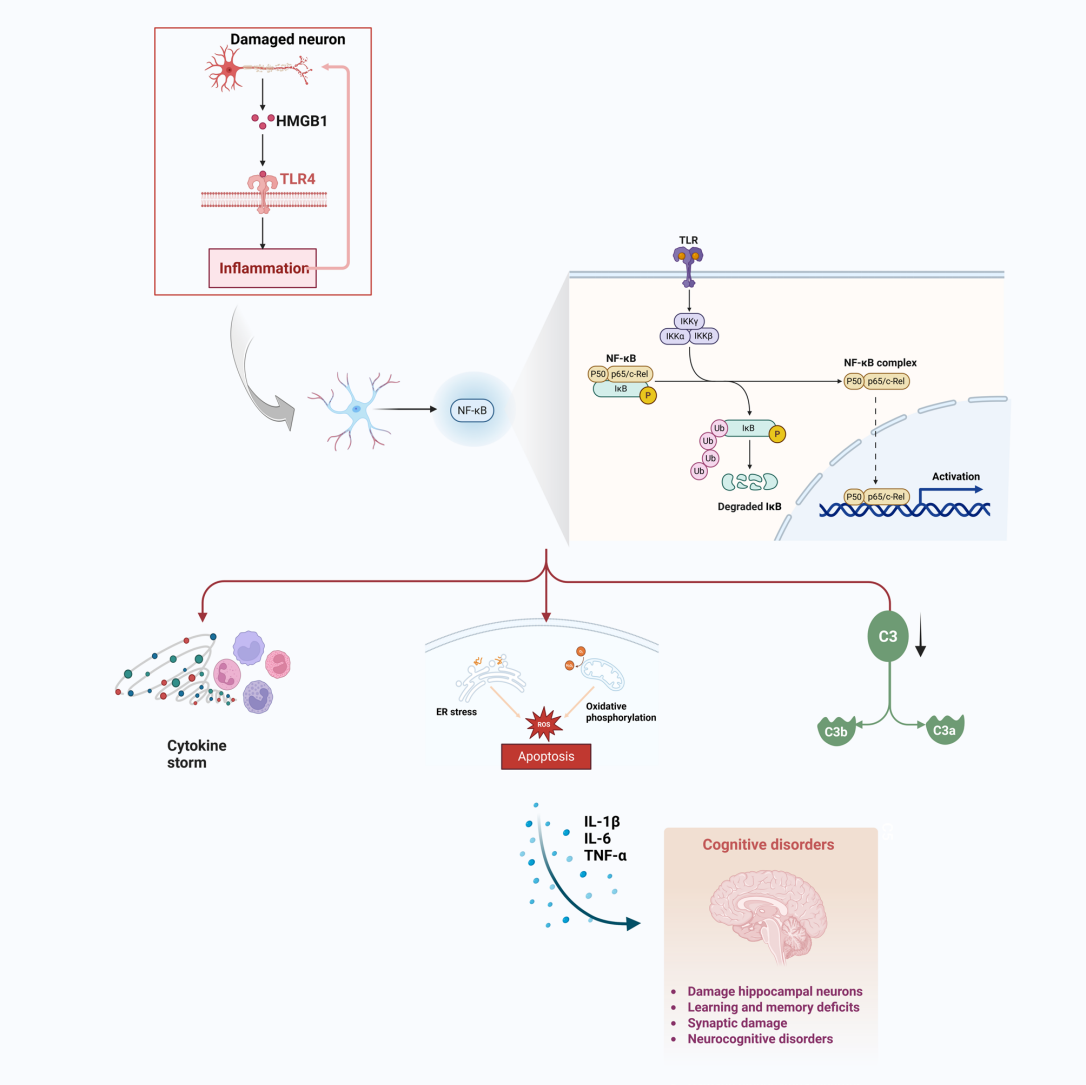
The NF-κB signaling pathway in astrocyte reactivity and the pathogenesis of perinatal brain injury. TLR4 typically expresses in the membrane of astrocytes, which can be activated by exogenous pathogen-associated molecular patterns (e.g., LPS) and endogenous injury-associated molecular patterns (e.g., HMGB1). When activated, it may recruit downstream proteins to induce mitochondrial assembly and NF-κB activation, thereby increasing the release of IL-1β and TNF-α. These inflammatory factors cause damage to nerve cells and initiate inflammatory cascade, which subsequently exacerbate inflammatory response, cell apoptosis, and oxidative stress in the brain. This can lead to cognitive impairment, incomplete hippocampal development, and learning and memory deficits. In addition, NF-κB pathway-induced pathologically remodeled astrocytes are found to damage hippocampal neurons by releasing complement component 3 (C3). *HMGB1*: high mobility group protein B; *TLR4*: toll-like receptor 4; *NF-κB* nuclear factor-kappa B; *IL-1β* interleukin-1β; *TNF-α* tumour necrosis factor alpha (Figure created with Biorender.com)

NF-κB is a widely expressed transcription factor that plays a crucial role in regulating immune and inflammatory responses. It binds to specific conserved DNA sequences to activate genes involved in inflammation, cell survival, and immune regulation. NF-κB is normally sequestered in the cytoplasm by inhibitory proteins (IκBs). Upon activation by stimuli such as cytokines, pathogens, or stress, IκBs are degraded, allowing NF-κB to translocate to the nucleus and activate target genes (Sun et al. 2022) (Fig. 2).

Dysregulation of the NF-κB pathway is implicated in perinatal brain injury. In fetal inflammatory response syndrome, the activation of nuclear NF-κB pathway proteins can trigger an inflammatory cascade that potentially leads to neurocognitive deficits, impaired hippocampal development, and learning and memory deficits (G. Singh, Segura, Georgieff, and Gisslen, 2021). HI injury induces rapid astrocytic upregulation of GFAP and pro-inflammatory cytokines (TNF-α, IL-6) alongside BBB disruption, a process strongly associated with NF-κB activation (Teo et al. 2023). Interventions targeting the NF-κB pathway have demonstrated protective effects against neonatal HI brain injury (Zhu et al. 2020). In a mouse model of stress, (Li, Wang, et al. 2020) further highlighted the role of NF-κB in neuroinflammation by showing that exosomal miR-207 alleviated depressive symptoms in stressed mice by targeting Tril to inhibit NF-κB signaling in astrocytes. These findings underscore the critical role of the NF-κB pathway in neuroinflammation and brain injury.

The NF-κB family consists of five transcription factors, including NF-κB1 (p105/p50), NF-κB2 (p100/p52), RelA (p65), RelB, and c-Rel. They can be activated by different ligands and are involved in signaling through the classical and nonclassical pathways (Yao et al. 2023). The classical pathway rapidly activates the inhibitor of the κB kinase (IKK) complex. The activated IKK then phosphorylates inhibitors of NF-κB (IκB) proteins and labels them for proteasomal ubiquitination and degradation, a process that releases p65/p50 and c-Rel/p50 complexes (Radzka et al., 2023). These complexes bind to homologous κB motifs in the nucleus, activating and promoting the expression of NF-κB target genes and regulating the transcription of genes containing shared κB-binding sites in their DNA enhancers or promoters, including genes encoding pro-inflammatory cytokines, chemokines, IκBα, and RelB (Yu, Lin, Zhang, Zhang, & Hu, 2020). The nonclassical pathway is activated by a subset of members of the tumor necrosis factor receptor superfamily, activating NF-κB-inducible kinase (NIK) (Chatterjee et al. 2019). NIK phosphorylates IκB kinase α, which produces p52 by phosphorylating the C-terminus of p100. During the development of immune cells, p52/RelB translocates to the nucleus after the phosphorylation cascade and activates the target genes (Peng, Ouyang, Lu, and Li, 2020).

NF-κB signaling in astrocytes is very important in CNS inflammation. Under normal conditions, cytoplasmic NF-κB is bound to its inhibitory protein κB (IκB) in an inactive state. Once activated, IκB is degraded and NF-κB is translocated to the nucleus to initiate the transcription of pro-inflammatory cytokines (Luo, Deng, Chen, Ding, & Li, 2023). In a mouse model with autoimmune encephalomyelitis, NF-κB pathway-induced pathologically remodeled astrocytes are found to damage hippocampal neurons by releasing complement component 3 (C3) (Hou et al. 2020). Meanwhile, interference with the NF-κB signaling pathway inhibits complement C3 and promotes astrocyte remodeling, thus attenuating synaptic damage and cognitive dysfunction (Jiang et al. 2023).

The HMGB1-TLR4-NF-κB signaling pathway is a critical inflammatory signaling pathway in perinatal brain injury. TLR4, widely expressed on astrocyte membranes, can be activated by both exogenous pathogen-associated molecular patterns (e.g., LPS) and endogenous damage-associated molecular patterns (e.g., HMGB1) (Gao et al. 2022). Upon activation, TLR4 recruits downstream proteins, leading to mitochondrial dysfunction and NF-κB activation, which subsequently stimulates the release of pro-inflammatory cytokines such as IL-1β and TNF-α. This cascade results in neuronal cell damage, inhibits neuronal regeneration and differentiation, and exacerbates neuroinflammation and oxidative stress (Abbaszadeh, Jorjani, Joghataei, Raminfard, & Mehrabi, 2023).

The HMGB1-TLR4 pathway plays a significant role in immature brain injury acquired through inflammation. Macrophages and damaged neurons release HMGB1, which activates astrocytes via the HMGB1/NF-κB signaling pathway. Reactive astrocytes further release HMGB1, creating a positive feedback loop that amplifies the inflammatory response, apoptosis, and oxidative stress in the brain (R. Fan, Wang, Botchway, Zhang, and Liu, 2022). Activation of this pathway in brain astrocytes leads to increased release of pro-inflammatory cytokines, contributing to neuronal injury and impaired recovery (Del Pozo et al., 2022; Wang et al. 2021a; Yang, Chan, Gao, Zheng, and Ke, 2023).

Moreover, the TLR4 pathway has been associated with HIE in neonates. HI triggers the release of HMGB1, which binds to TLR4 on astrocytes and microglia, leading to NF-κB activation and subsequent neuroinflammation. This process is associated with the pathogenesis of HIE, as it exacerbates brain injury by promoting cytokine release and neuronal cell death (Kremsky et al. 2023; Wu et al. 2019). Additionally, TLR4 activation has been shown to impair neurogenesis and synaptic plasticity, contributing to long-term neurological deficits in affected infants. The activation of HMGB1-TLR4-NF-κB pathway leads to a cascade of inflammatory responses, oxidative stress, and impaired neuronal repair, highlighting its potential as a therapeutic target for mitigating brain injury in neonates.

Emerging evidence suggests that activation of the HMGB1/TLR4/NF-κB pathway in the developing neonatal brain induces persistent epigenetic modifications that may underlie long-term neurodevelopmental impairments. We hypothesize that HMGB1-mediated TLR4 activation triggers widespread epigenetic reprogramming through: DNA hypermethylation of neuroprotective genes via upregulation of DNMTs (Fan et al. 2025), histone modifications associated with neuroinflammation (Escartin et al. 2021), and DNA methylation-dependent silencing of neurodevelopmental regulators (Cuautle et al. 2024). These modifications could establish a self-perpetuating cycle of neuroinflammation by locking astrocytes into a chronically reactive state, as demonstrated by sustained IL-1β/TNF-α expression in LPS-exposed neonatal astrocytes (Avendaño, Montero, Chávez, von Bernhardi, and Orellana, 2015). Moreover, HMGB1-induced epigenetic silencing of synaptic plasticity genes via H3K9me3 may explain persistent cognitive deficits (Shentu et al. 2024). Therapeutically, targeting these epigenetic mechanisms may reverse pathological reprogramming and restore neurodevelopmental trajectories.

Emerging evidence underscores the critical role of HMGB1-TLR4-NF-κB signaling in epileptogenesis, linking perinatal brain injury to the development of epilepsy later in life. Clinical studies suggest that inflammatory insults during the neonatal period, such as HIE, prime the brain for hyperexcitability and spontaneous seizures by perpetuating neuroinflammation and astrocyte dysfunction (Hanin et al. 2023). HMGB1, released by damaged neurons and activated microglia, binds to TLR4 on astrocytes, triggering NF-κB-driven pro-inflammatory cytokine release and disrupting glutamate homeostasis—a key mechanism in epileptic circuitry (Paudel et al. 2018). Reactive astrocytes, through persistent HMGB1-TLR4 activation, foster a vicious cycle of inflammation, synaptic rewiring, and aberrant neurogenesis, contributing to temporal lobe epilepsy and refractory seizures in adulthood (Rosciszewski et al. 2019). Notably, astrocytic TLR4 knockdown attenuates seizure severity in experimental models, highlighting its therapeutic potential (Chang et al. 2022). These findings position perinatal HMGB1-TLR4 dysregulation as a latent driver of epileptogenesis, with astrocytes serving as pivotal mediators between early-life injury and chronic neurological sequelae.

### Notch signaling pathway

The Notch signaling pathway refers to a class of highly conserved cell surface receptors encoded by Notch genes that mediate intercellular signaling through receptor-ligand interactions. The Notch signaling pathway comprises five Notch ligands (Jagged1 and 2, DLL1, 3, and 4), four Notch receptors (Notch1 ~ 4), CSL proteins, and Notch effectors (Weinmaster and Kintner 2003). Notch1 receptor and Jagged1 ligand are upregulated following brain injury, activate astrocyte signaling and regulate astrocyte regeneration. In mammals, Notch signaling is activated by binding of Notch receptors (Notch1-4) to ligands (e.g., Jagged1). Subsequently, this complex is cleaved by γ-secretase, leading to cleavage of the negative regulatory region protein and the release of the Notch intracellular domain (NICD). NICD is transported to the nucleus, where it interacts with the CSL intranuclear transcription factor to form the transcriptional activation complex NICD/CSL. This complex is recognized by the mastermind-like protein, recruiting co-activators that assemble an active transcriptional complex at the target promoter to activate the transcription and expression of target genes encoding Hes, Hey, and other members of the basic–helix–loop–helix transcription repressor family, thus regulating cell fate and functions (Shim, Lee, and Hwang, 2022; Zhang et al. 2023). Furthermore, the Notch signaling pathway interacts with Hedgehog, Wnt, and other signaling pathways associated with astrocyte proliferation, migration, and maturation (Yang and Jackson 2019).

**Fig. 3:**
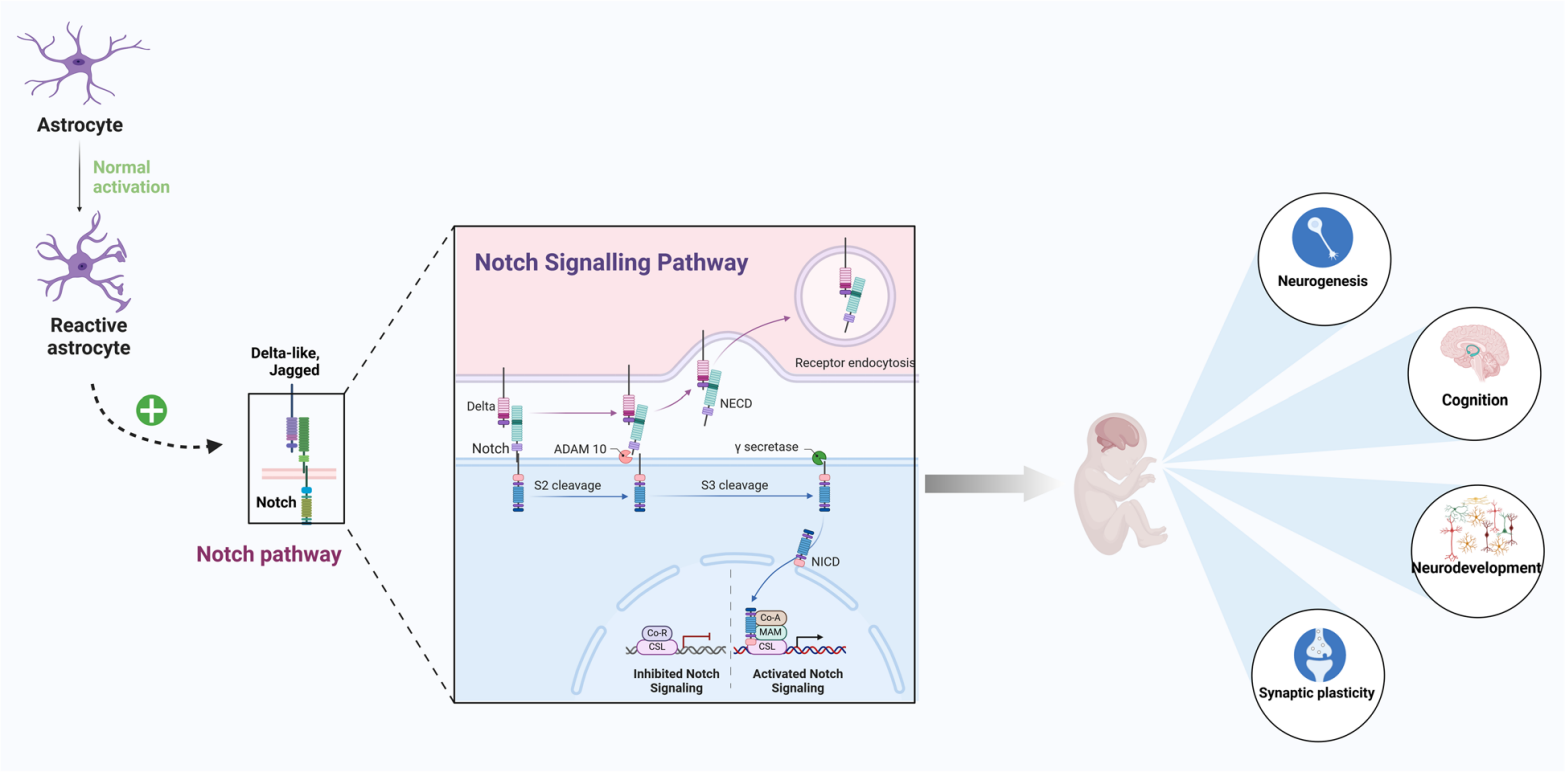
The Notch signaling pathway in astrocyte reactivity and the pathogenesis of perinatal brain injury. Under normal conditions, Notch activation promotes astrocyte differentiation, modulates their morphology, and regulates inflammatory responses. It also supports neurogenesis in the striatum, enhances synaptic plasticity, and influences neurocognitive functions, including hippocampal-dependent learning and memory (Figure created with Biorender.com)

The Notch signaling pathway helps maintain homeostasis in embryonic and postnatal development across species. During embryonic development, the Notch pathway ensures the proportional proliferation of neurons and glial cells and is particularly active in astrocytes. Its downregulation is essential for initiating neurogenic programs necessary for the development of the nervous system (Baines et al. 2020; Ma, Kutchy, Chen, Meigs, & Hu, 2022), (Liu et al. 2021) demonstrated in a rat model of generalized anxiety disorder that downregulation of the Notch signaling pathway promoted hippocampal neuron regeneration, thereby alleviating anxiety-like behaviors. In neonatal rats, LPS injection was shown to inhibit neurogenesis by activating the Notch signaling pathway, leading to late-onset cognitive dysfunction (Fig. 3). This highlights the dual role of the Notch pathway: while its proper regulation is crucial for normal neurodevelopment, its dysregulation-whether through overactivation or suppression-may contribute to neurodevelopmental disorders and cognitive impairments.

Notch signaling promotes astrocyte differentiation and modulates reactive astrocyte morphology and their response to inflammatory stimuli. In murine studies, the Notch signaling pathway triggers the neurogenesis of astrocytes in the striatum and affects neurocognitive functions such as learning and memory (Huang et al. 2021; Santopolo, Magnusson, Lindvall, Kokaia, and Frisén, 2020). In the spinal cord of rats with paclitaxel-induced neuropathic pain, it was found that both pathologically remodeled astrocytes and Notch signaling were significantly activated and that molecules downstream of Notch signaling were co-localized with pathologically remodeled astrocytes (Li et al. 2022a). This process may be the result of activated Notch1/Jagged1 signaling, which promotes Notch1 cleavage and translocation of NICD1 into the nuclei of reactive cortical astrocytes (Ribeiro et al. 2021). Furthermore, in neonatal hypoxic–ischemic injury, the Notch pathway regulates glutamate transport in astrocytes, which subsequently modulates synaptic plasticity by suppressing autophagy (Li, Lu, Cui, Wang and Zheng, 2023a; Li, Lu, Cui, Wang and Y. Zheng, 2023b). These results suggest that astrocyte-mediated Notch signaling pathway is integral to the onset and development of perinatal brain injury. However, the Notch signaling pathway has been investigated primarily by animal experiments. More pre-clinical studies are required to elucidate its mechanism.

### High-affinity excitatory glutamate transporter signaling pathway

**Fig. 4:**
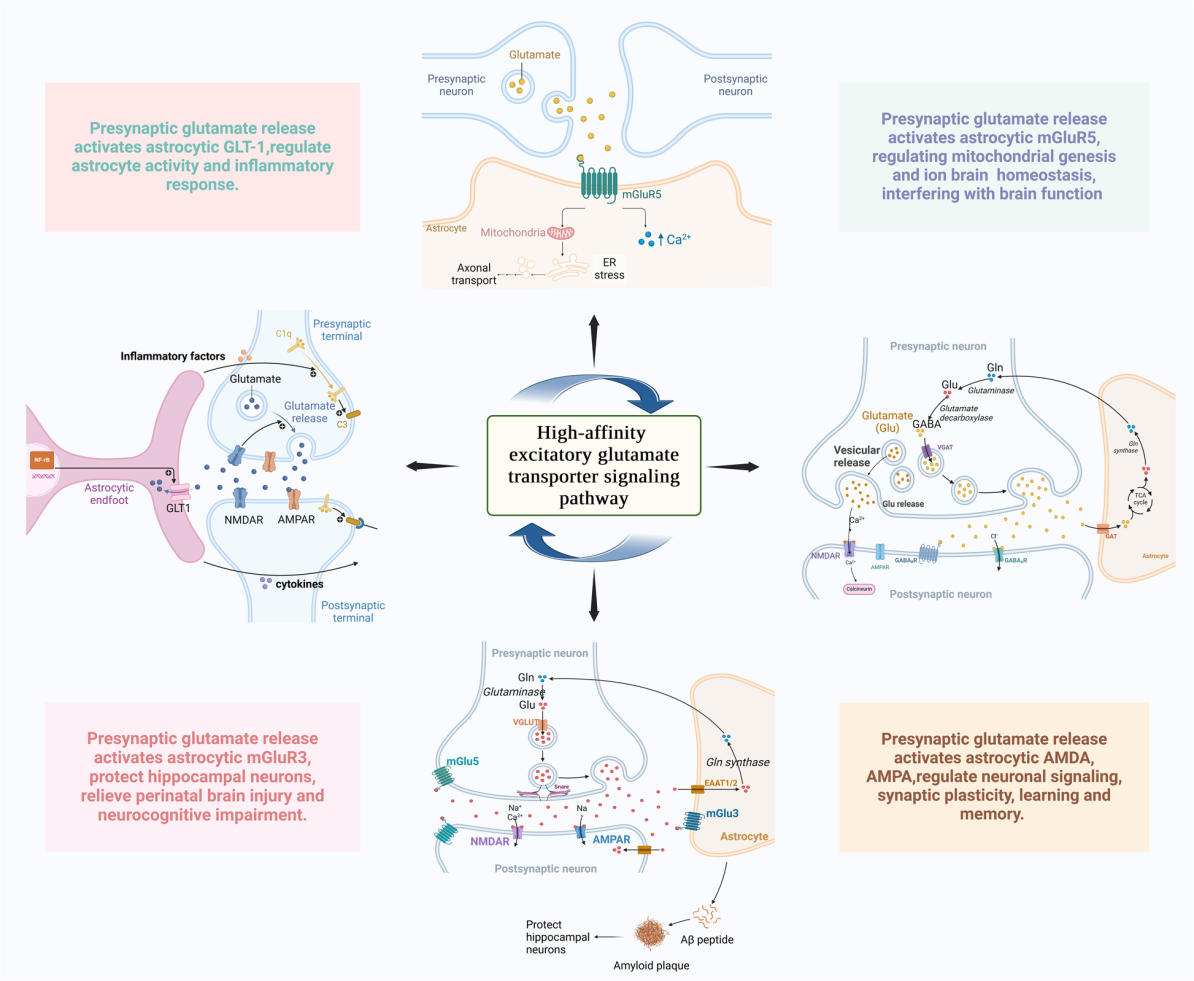
The High-affinity excitatory glutamate transporter signaling pathway in astrocyte reactivity of perinatal brain injury. Astrocytes can mediate vesicular release of glutamate and regulate white matter. Dynamic induction of NF-κB by GLT-1 in astrocytes regulates astrocyte activity; Environmental glutamate activates GABA interneurons through high-affinity NMDA receptors, regulating neuronal signal transduction and ion homeostasis; The glutamate receptors mGluR5 and mGluR3 are widely expressed in astrocytes during development, which can regulate the biogenesis of astrocyte mitochondria and synaptic plasticity in the brain. *NF-κB* nuclear factor-kappa B; *GLT-1* glutamate transporter-1; *GABA* γ-aminobutyric acid; *NMDA* n-methyl D-aspartate receptor; *mGluR5* metabotropic glutamate receptor 5; *mGluR3* metabotropic glutamate receptor 3; *AMPARs* AMPA-type glutamate receptors (Figure created with Biorender.com)

Glutamate is the predominant excitatory neurotransmitter in the CNS. Its activity depends on the expression of α-amino-3-hydroxy-5-methyl-4-isoxazolepropionic acid and N-methyl-D-aspartate (NMDA) and excitatory aminotransporter proteins in neurons and glial cells. Glutamate initiates rapid signaling in the synapse and is closely related to learning and memory (Li, Lu, Cui, Wang and Zheng, 2022a). Chemokine and neuropeptide in astrocytes mediate glutamate release from vesicles to regulate white matter development. For a developing brain, regulation of the number and function of synapses contributes to the reordering and refinement of neural connections and may be critical for brain repair, sensory restoration, and restoration from neurodevelopmental disorders (Martínez-Gallego, Pérez-Rodríguez, Coatl-Cuaya, Flores, and Rodríguez-Moreno, 2022) (Fig. 4). Platelet-reactive proteins secreted by astrocytes have been shown to induce the formation of structural synapses (Fossati et al., 2019). In addition, glutamate-amino acid aspartate transporter protein, GLT-1 and their human homologs excitatory amino acid transporter proteins 1 and 2 may prevent synaptic signaling abnormalities, excitotoxicity, or cell death resulting from glutamate overaccumulation at synapses. This may significantly influence glial- and axonal-mediated pre-myelinic white matter damage during periventricular leukomalacia and cerebral palsy (Chang et al., 2021; Lee et al. 2022; Pajarillo, Rizor, Lee, Aschner, and Lee, 2019).

GLT-1 is the predominant amino transporter protein of glutamate in the CNS, which controls astrocyte activation and inflammatory responses (Cheng et al. 2020 Zhang, Chu, and Wong, 2022). Impaired mouse astrocyte ctivation is associated with strongly downregulated astrocyte GLT-1, which reduces glutamate re-uptake of astrocytes and modulates glutamatergic synaptic function (Lu et al. 2020). The dynamic induction of GLT-1 is also dependent on the presence of neurons in astrocytes and the activation is dependent on neuronal activity of NF-κB (Dresselhaus & Meffert 2019). Depressive-like behaviors can be modulated by acting on GLT-1 synaptic transmission (X. Liu et al. 2022).

In the cerebral cortex, glutamate activates NMDA receptors in noradrenergic neurons to regulate glutamatergic transmission, long-term plasticity, and astrocyte proliferation. Environmental glutamate, which refers to extracellular glutamate from non-synaptic sources such as glial release or pathological conditions, plays a critical role in neonatal brain development. It activates immature GABAergic interneurons via high-affinity NMDA receptors, which is essential for establishing normal cortical inhibition (Quintas, Gonçalves, and Queiroz, 2023; J. Zhou et al. 2022). However, in HIE, HI induces ATP depletion, leading to energy failure,which impairs astrocytic glutamate transporters (GLT-1/EAAT2 and GLAST/EAAT1) that normally maintain extracellular glutamate homeostasis. The energy failure causes loss of glutamate uptake capacity due to disrupted Na + /K + gradients, reversed transporter operation releasing glutamate, and neuronal depolarization enhancing synaptic glutamate release—collectively creating an excitotoxic glutamate surge that overactivates NMDA receptors, leading to Ca2 + overload and neuronal death (Thapaliya, Pape, Rose, and Ullah, 2023). Astrocytes mediate this process through reducing glutamate uptake (via downregulated GLAST/GLT-1 transporters), dysregulating calcium signaling and the release of pro-inflammatory cytokines, thereby exacerbating neuronal damage. Thus, astrocytes play a central role in both normal glutamate metabolism and pathological mechanisms of excitotoxicity in perinatal brain injury (Hanson et al. 2019; Pál, 2018).

Astrocytes widely express glutamate receptors (mGluR5 and mGluR3), which may contribute to the maturation of excitatory synapses (Kellner et al. 2021). Metabotropic glutamate receptor 5 (mGluR5) is abundant in neonatal astrocytes. It regulates the expression and function of the glutamate transporter protein, as well as hippocampal excitatory signaling, dynamically controlling synaptic plasticity and interfering with continuous brain function (Danjo et al., 2022; Tanaka et al. 2021; Umpierre, West, White and Wilcox, 2019). mGluR5 controls the metabolic regulator peroxisome proliferator-activated receptor and coactivator 1α, regulates astrocyte mitochondrial biogenesis, inhibits astrocyte morphogenesis, and affects the formation and function of neighboring synapses (Zehnder et al. 2021). Additionally, activated metabotropic glutamate receptors (mGlu3R) in astrocytes protect hippocampal neurons against Aβ neurotoxicity by stimulating neurotrophin release and Aβ uptake, which helps prevent inflammation-induced perinatal brain injury (Turati et al. 2022; Zinni et al. 2021). Importantly, interactions between mGlu3 and mGlu5 receptors are also involved in mechanisms of neuronal toxicity. mGlu3 receptors are conventionally associated with the control of neurotransmitter release. They support the signaling of the mGlu5 receptor in neurons and mediate excitatory amino acid-induced PI hydrolysis in the developing brain. Furthermore, mGlu3 receptors can modulate synaptic plasticity in regions of the brain that shows significant mGlu5-mediated plasticity (Di Menna et al. 2018).

## Crosstalk between astrocytes and microglia, oligodendrocytes, and neurons in perinatal brain injury

**Fig. 5:**
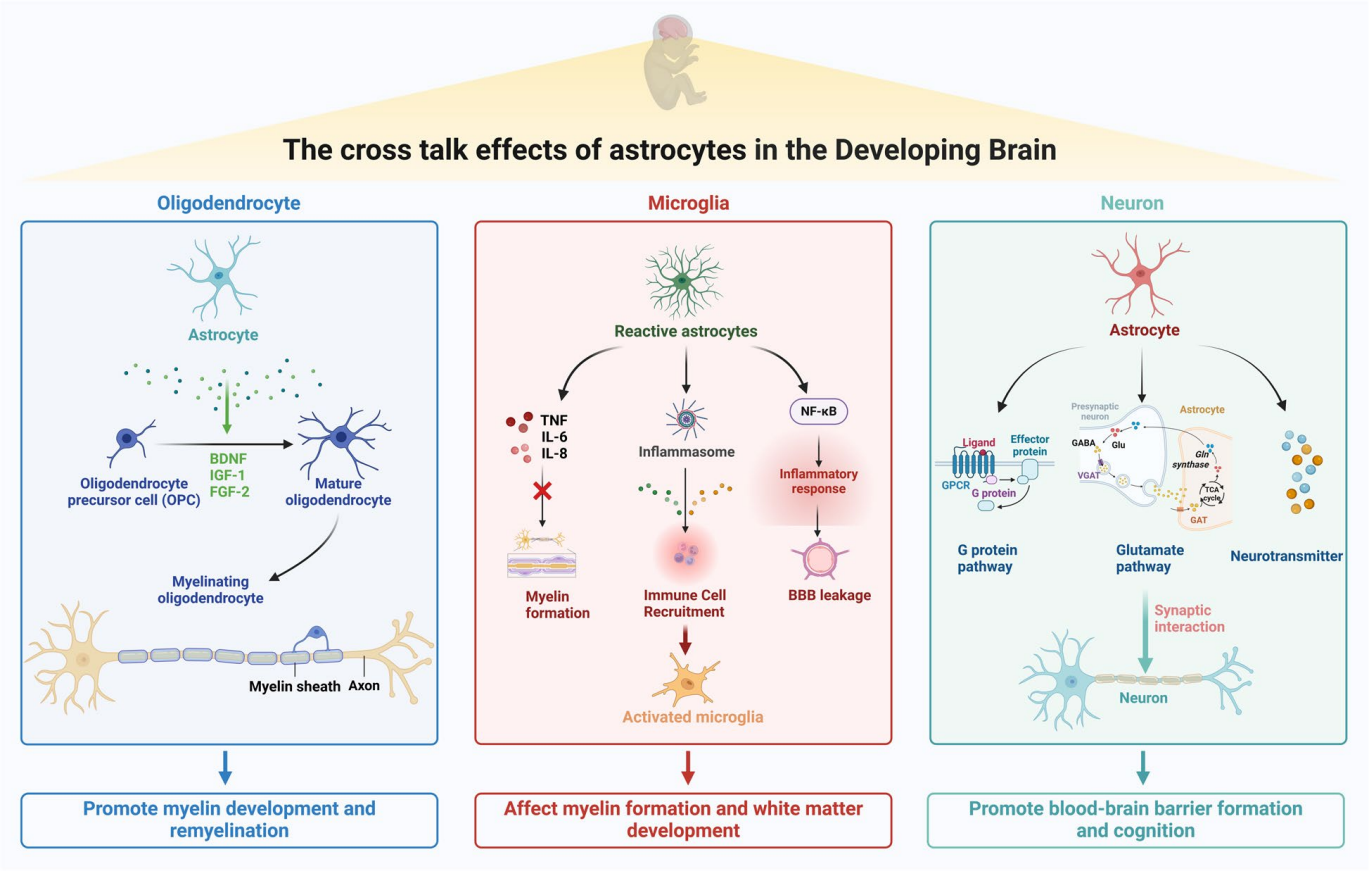
Crosstalk mechanisms between astrocytes and microglia, oligodendrocytes, and neurons in the perinatal brain injury. Astrocytes play a crucial role in mediating interactions among microglia, oligodendrocytes, and neurons. Through the NF-κB pathway, astrocytes establish close contact with microglia at synapses, regulating neuroinflammation and maintaining neuronal function. Astrocytes secrete growth and nutritional factors to promote oligodendrocyte activation and enhance myelination, while downregulating the Nrf2 pathway to support oligodendrocyte survival and white matter development. Additionally, astrocytes communicate with neurons by releasing chemical signals and expressing neurotransmitter receptors, promoting cognitive functions and blood–brain barrier integrity; *TNF* tumour necrosis factor; *NF-κB* nuclear factor-kappa B; *BBB* blood–brain barrier (Figure created with Biorender.com)

Astrocytes are the major homeostatic regulators of the CNS, providing metabolic substrates to neurons and other neuroglia, regulating ions and neurotransmitters in the extracellular space. Additionally, astrocytes modulate immediate communications between microglia, oligodendrocytes, and neurons, which are essential for immediate responses to microenvironmental changes, serving a critical role in the pathways in perinatal brain injury (Fig. 5).

### Astrocyte-microglia crosstalk

Microglia and astrocytes are key glial cells in the CNS that interact closely to regulate brain homeostasis, immune responses, and synaptic function. Microglia and astrocytes support neuronal and BBB functions, engage in dynamic crosstalk to modulate synaptic activity and neuroinflammation (Rangel-Gomez et al., 2024). Astrocytes establish close contact with microglia at synapses, providing signals to influence microglial activity and synaptic pruning. Additionally, astrocytes regulate axon growth via NF-κB signaling, while microglia respond to these signals to maintain tissue integrity and repair (S. Yang et al., 2021). This interplay between microglia and astrocytes is crucial for both normal brain function and the response to injury or disease. In a mouse model with traumatic brain injury (TBI), the astrocyte-derived exosome-miR-873a-5p was found to attenuate microglia-mediated neuroinflammation and improve neurological deficits by inhibiting the NF-κB signaling pathway (Long et al. 2020). Additionally, astrocytes are also source of IL-17, which interacts with TNF-α and leads to neutrophil infiltration and expression of pro-inflammatory molecules. This further activates the microglial M1 phenotype, thereby inducing neurotransmitter metabolism and depression-like behaviors (C. Huang, Zhang, Li, and Song, 2022).

M2 microglia are anti-inflammatory and neuroprotective phenotypes that play crucial roles in tissue repair and homeostasis. M2 microglia promote resolution of inflammation, clear cellular debris, and support tissue regeneration. Their crosstalk is essential for mitigating neuroinflammation and promoting recovery after injury (Kim & Son, 2021). Studies have shown that lipid-rich reactive astrocytes stimulate neuronal oxidation and oxidative stress, activate microglia through IL-3 signaling, and inhibit myelin biosynthesis, intervening in the development of brain white matter in preterm infants (He et al. 2022; Mi et al. 2023). In turn, in microglia, the NF-κB pathway activates NLRP3 inflammatory vesicles, which then activate cystatinase-1 to induce the secretion of A1-inducing factor, leading to the generation of pathologically remodeled astrocytes (S. Li et al., 2022c). Furthermore, depletion of microglia alters astrocyte proliferation and scar formation and induces inflammation spreading, partly by inhibiting STAT3 phosphorylation in astrocytes after spinal cord injury (Z. L. Zhou et al. 2023).

### Effects of astrocytes on oligodendrocyte progenitor cell maturation and myelin defects in perinatal brain injury

Recent studies have shown that astrocytes play a crucial role in the survival, proliferation, and differentiation of oligodendrocyte progenitor cells (OPCs), which are essential for generating mature oligodendrocytes. These mature oligodendrocytes are responsible for developing and maintaining myelin sheaths, as well as facilitating remyelination after perinatal injury (Singh et al. 2018). Astrocytes secrete growth and trophic factors, such as BDNF, ciliary neurotrophic factor (CNTF), insulin-like growth factor 1 (IGF-1), fibroblast growth factor-2 (FGF-2), and leukemia inhibitory factor (LIF), which promote the survival and differentiation of OPCs into mature oligodendrocytes, thereby enhancing myelin formation (Kakae et al., 2023; Koizumi 2021). Additionally, astrocyte-mediated downregulation of Nrf2 promotes oligodendrocyte regeneration by enhancing cholesterol biosynthesis (via HMGCS1/FDPS upregulation) and releasing semaphorin 3a/6a to facilitate OPC detachment from inhibitory endothelial niche (Chen et al. 2024; Molina-Gonzalez et al. 2023).

### Effects of astrocytes on neurons in perinatal brain injury

Astrocytes can make contact with neuronal synapses and form end feet that wrap around capillaries, serving as key components of the BBB. Furthermore, astrocytes can respond to synaptic activity by releasing chemical signals (gliotransmitters) and expressing neurotransmitter receptors (G-protein-coupled and ionotropic receptors), thus communicating with neurons (Cervetto et al. 2023). Neuron-astrocyte crosstalk at tripartite synapses is essential for cognition and that bidirectional glutamatergic communication between astrocytes and neurons facilitates the processing of enriched information in neuronal networks by regulating complex, spatially distributed synaptic signals (Bauminger and Gaisler-Salomon, 2022). Neurons can drive astrocyte maturation and promote the expression and output of astrocyte metabolic genes through Notch signaling, a process involving motions of multiple molecules through specific transporter proteins (Hasel, Aisenberg, Bennett, & Liddelow, 2023). During perinatal brain development, neuron-derived ligands interact with astrocyte Notch3 or gliadin 4, controlling astrocyte differentiation and morphogenesis, and upregulating astrocyte GLT-1 expression (Men et al. 2019).

### Metabolic dysregulation in astrocyte-neuron-oligodendrocyte crosstalk during perinatal brain injury

Astrocytes serve as central metabolic hubs in the developing brain, with their lactate shuttle system playing a pivotal role in maintaining energy homeostasis for both neurons and oligodendrocytes. Under pathological conditions, reactive astrocytes undergo significant alterations in their metabolic programming, downregulating critical homeostatic genes including monocarboxylate transporters, glutamine synthetase, and glucose transporters (Leng et al. 2022). This metabolic shift disrupts the astrocyte-neuron lactate shuttle, depriving neurons of essential energy substrates during periods of high synaptic activity and potentially exacerbating excitotoxic injury (Magistretti & Allaman 2018). Similarly, OPCs, which exhibit high metabolic demands during differentiation, become particularly vulnerable to this energy deficit, leading to impaired maturation and subsequent myelination defects (Rinholm et al. 2011). We hypothesize that this early disruption of metabolic coupling creates a self-perpetuating cycle: impaired lactate supply compromises neuronal activity and OPC differentiation, which in turn reduces neuronal signaling necessary for maintaining astrocyte homeostasis (Rothman et al. 2022). This triad of dysfunction may underlie the long-term cognitive and motor deficits observed in perinatal brain injury survivors, suggesting that therapeutic strategies targeting astrocyte metabolic recovery could simultaneously benefit multiple neural cell populations and improve overall brain network function.

## Concluding remarks and future perspective

Astrocytes play an important role in the pathogenesis of perinatal brain injury, by regulating neuroinflammation, synaptic function, and cellular crosstalk through signaling pathways such as JAK/STAT, NF-κB, Notch, and glutamate transporters. These pathways mediate BBB disruption, reactive astrocyte proliferation, and excitotoxicity, while interactions with microglia, oligodendrocytes, and neurons may further shape the injury response. The molecular mechanisms remain incompletely understood. Future research, utilizing both animal models and human tissue, should focus on identifying region- and time-specific processes to develop targeted therapies for perinatal brain injury.

## Data Availability

No datasets were generated or analysed during the current study.

## Abbreviations

CNS: Central nervous system
TGF-β: Transforming growth factor-β
VEGF: Vascular endothelial growth factor
GDNF: Glial cell-derived neurotrophic factor
BDNF: Brain-derived neurotrophic factor
BBB: Blood–brain barrier
HI: Hypoxic-ischemic
NF-κB: Nuclear factor-kappa B
JAK/STAT: Janus kinase/signal transducers and activators of transcription
SVZ: Subventricular zone
GFAP: Glial fibrillary acidic protein
HIE: Hypoxic-ischemic encephalopathy
LPS: Lipopolysaccharide
NICD: Notch intracellular domain
NMDA: N-methyl-D-aspartate
GLT-1: Glutamate transporter protein 1
mGluR5: Metabotropic glutamate receptor 5
GLAST: Glutamate-aspartate transporter
NIK: NF-κB-inducible kinase
TBI: Traumatic brain injury
OPCs: Oligodendrocyte progenitor cells
CNTF: Ciliary neurotrophic factor
IGF-1: Insulin-like growth factor 1
FGF-2: Fibroblast growth factor-2
LIF: Leukemia inhibitory factor
